# Assessment of molecular markers for anti-malarial drug resistance after the introduction and scale-up of malaria control interventions in western Kenya

**DOI:** 10.1186/s12936-015-0588-4

**Published:** 2015-02-14

**Authors:** Monica Shah, Yusuf Omosun, Ashima Lal, Christopher Odero, Wangeci Gatei, Kephas Otieno, John E Gimnig, Feiko ter Kuile, William A Hawley, Bernard Nahlen, Simon Kariuki, Edward Walker, Laurence Slutsker, Mary Hamel, Ya Ping Shi

**Affiliations:** Malaria Branch and Division of Parasitic Diseases and Malaria, Center for Global Health, Centers for Disease Control and Prevention, Atlanta, GA USA; Atlanta Research and Education Foundation, Atlanta, GA USA; Center for Global Health Research, Kenya Medical Research Institute, Kisumu, Kenya; Liverpool School of Tropical Medicine, Liverpool, UK; UNICEF, Child Survival and Development Cluster, Jakarta, Indonesia; President’s Malaria Initiative, Washington, DC USA; Michigan State University, East Lansing, USA

**Keywords:** Anti-malarial drug resistance, ITNs, Chloroquine resistance, Sulphadoxine-pyrimethamine resistance, ACT resistance, Vector control

## Abstract

**Background:**

Although it is well known that drug pressure selects for drug-resistant parasites, the role of transmission reduction by insecticide-treated bed nets (ITNs) on drug resistance remains unclear. In this study, the drug resistance profile of current and previous first-line anti-malarials in Kenya was assessed within the context of drug policy change and scale-up of ITNs. National first-line treatment changed from chloroquine (CQ) to sulphadoxine-pyrimethamine (SP) in 1998 and to artemether-lumefantrine (AL) in 2004. ITN use was scaled-up in the Asembo, Gem and Karemo areas of western Kenya in 1997, 1999 and 2006, respectively.

**Methods:**

Smear-positive samples (N = 253) collected from a 2007 cross-sectional survey among children in Asembo, Gem and Karemo were genotyped for mutations in *pfcrt* and *pfmdr1* (CQ), *dhfr* and *dhps* (SP)*,* and at *pfmdr-*N86 and the gene copy number in *pfmdr1* (lumefantrine). Results were compared among the three geographic areas in 2007 and to retrospective molecular data from children in Asembo in 2001.

**Results:**

In 2007, 69 and 85% of samples harboured the *pfmdr1*-86**Y** mutation and *dhfr/dhps* quintuple mutant, respectively, with no significant differences by study area. However, the prevalence of the *pfcrt*-76**T** mutation differed significantly among areas (p <0.02), between 76 and 94%, with the highest prevalence in Asembo. Several 2007 samples carried mutations at *dhfr*-164**L**, *dhps*-436**A**, or *dhps*-613**T**. From 2001 to 2007, there were significant increases in the *pfcrt*-76**T** mutation from 82 to 94% (p <0.03), *dhfr/dhps* quintuple mutant from 62 to 82% (p <0.03), and an increase in the septuple CQ and SP combined mutant haplotype, **K**_**76**_**Y**_**86**_**I**_**51**_**R**_**59**_**N**_**108**_**G**_**437**_**E**_**540**_, from 28 to 39%. The prevalence of the *pfmdr1*-86**Y** mutation remained unchanged. All samples were single copy for *pfmdr1*.

**Conclusions:**

Molecular markers associated with lumefantrine resistance were not detected in 2007. More recent samples will be needed to detect any selective effects by AL. The prevalence of CQ and SP resistance markers increased from 2001 to 2007 in the absence of changes in transmission intensity. In 2007, only the prevalence of *pfcrt*-76**T** mutation differed among study areas of varying transmission intensity. Resistant parasites were most likely selected by sustained drug pressure from the continued use of CQ, SP, and mechanistically similar drugs, such as amodiaquine and cotrimoxazole. There was no clear evidence that differences in transmission intensity, as a result of ITN scale-up, influenced the prevalence of drug resistance molecular markers.

**Electronic supplementary material:**

The online version of this article (doi:10.1186/s12936-015-0588-4) contains supplementary material, which is available to authorized users.

## Background

Case management of malaria currently relies heavily on the use of a limited set of effective anti-malarial drugs which may become compromised by the development and spread of drug resistance [1]. Parasite resistance to anti-malarial drugs is driven mainly by drug pressure but it has been hypothesized that changes in malaria transmission intensity due to the scale up of vector control interventions, such as insecticide-treated bed nets (ITNs), could also affect anti-malarial drug resistance [2]. In areas where drug policy has changed and transmission-reducing malaria control interventions have achieved high coverage, monitoring the prevalence of molecular markers for drug resistance to current treatment drugs and to previously used drugs could provide a better understanding of how changes in drug pressure and the intensity of malaria transmission influence the profile of molecular markers for anti-malarial drug resistance.

The role of drug pressure on the emergence of parasite resistance to anti-malarials has been well described [3]. Due to widespread parasite resistance to chloroquine (CQ) and, subsequently, sulphadoxine-pyrimethamine (SP), all malaria-endemic countries in sub-Saharan Africa have adopted artemisinin-based combination therapy (ACT) as the first-line policy for treatment of uncomplicated *Plasmodium falciparum* infection [4]. However, the emergence of reduced sensitivity to artemisinins in focal areas of Southeast Asia has prompted global concern [5-10]. Although artemisinin resistance has not yet been documented on the African continent [11], monitoring parasite resistance to artemisinins or to ACT partner drugs is vital for malaria control. In addition, long-term monitoring of parasite sensitivity to previously withdrawn anti-malarial drugs, such as CQ, can provide useful surveillance information if these drugs target similar resistance markers to current or candidate ACT partner drugs [12].

In addition to drug pressure, many other factors can contribute to the development and spread of drug resistance, notably the pharmacodynamic properties of anti-malarials, substandard drugs, inadequate dosing, incomplete compliance, as well as human host factors such as age/immunity, vector and ecological characteristics [3]. Among these factors, the relationship between reductions in malaria transmission intensity by vector control tools and drug resistance is complex and remains unclear due to the limited empirical evidence available from field studies [13]. In Tanzania, the short-term use of ITNs was linked to decreased prevalence of a molecular marker associated with resistance to SP [14] and, in Zimbabwe, the implementation of indoor residual spraying (IRS) was associated with a lower risk of CQ treatment failure and reduced prevalence of CQ-resistant parasites [15]. However, evaluations of insecticide-treated curtains in Burkina Faso [16] and ITNs in western Kenya five years after their introduction indicated no clear effect of transmission reduction on the prevalence of resistance markers for SP and CQ [17]. Despite these conflicting findings, these studies suggest that changes in malaria transmission by vector control interventions, which are key elements of malaria control, might influence anti-malarial drug resistance.

Molecular markers are useful for monitoring parasite resistance to anti-malarial drugs. The combination of the dihydrofolate reductase (*dhfr*) triple mutant (S108**T**/N51**I/**C59**R**) with the dihydropteroate synthase (*dhps*) double mutant (A437**G**/K540**E**) has been associated with *in vivo* SP treatment failure [18]. Several single nucleotide polymorphisms (SNPs) in *P. falciparum* CQ transporter (*pfcrt)* gene (C72**S**, M74**I**, N75**E**, N75**K** and K76**T**) and *P. falciparum* multidrug-resistance 1 (*pfmdr1*) gene (N86**Y**, S1034**C**, N1042**D**, and D1246**Y**) have been linked to CQ resistance [19]. Among these SNPs, K76**T** and N86**Y** remain the most important [19,20]. *Pfcrt*-76**T** has also been associated with *in vivo* resistance to amodiaquine (AQ), a 4-aminoquinolone anti-malarial drug that is both structurally related to and shares a similar mechanism of action to CQ [21-23]. In addition, increases in the *pfmdr1* copy number have been associated with *in vivo* and/or *in vitro* decreased sensitivity to mefloquine and lumefantrine, the ACT partner drug in artesunate-mefloquine (AM) and the artemether-lumefantrine (AL), respectively [24-26]. Several *in vitro* and *in vivo* studies, conducted mainly in Africa, have shown that the selection of *pfmdr1-*N86, N1042, D1246 and *pfcrt-*K76 susceptible alleles and mutant type of *pfmdr1-*184**F** was associated with the development of parasite resistance to lumefantrine [27-32]. Collectively, these findings suggest that the detection of *pfmdr1* copy number and/or *pfmdr1* SNPs and *pfcrt*-K76 wild type could be useful molecular markers to monitor resistance to mefloquine and lumefantrine.

As part of a 2007 annual malaria survey conducted in three areas of western Kenya, this study assessed the prevalence of resistance markers to currently and previously used first-line anti-malarials within the context of drug policy change and scale-up of ITNs. The specific objectives were to: 1) determine the prevalence of drug-resistant molecular markers for CQ, SP and lumefantrine after the introduction of AL; 2) assess the relationship between the observed prevalence of drug resistance markers and differences in transmission intensity, as a result of ITN scale-up, among three geographically contiguous study areas in 2007; and, 3) compare the prevalence of the molecular markers from a subset of 2007 samples with molecular data from a 2001 survey from the same study area [17].

## Methods

### Study sites, population and design

This study was integrated into a 2007 annual malaria parasitaemia and anaemia cross-sectional survey conducted by the Kenya Medical Research Institute (KEMRI) and the US Centers for Disease Control and Prevention (CDC) in three contiguous areas in western Kenya: the Asembo area (formerly Rarieda Division, Bondo District), Gem (formerly Yala and Wagai Divisions, Siaya District) and Karemo (formerly Karemo Division, Siaya District) [33]. One of the primary aims of the survey was to evaluate the effect of malaria control interventions on the community-level prevalence of malaria parasitaemia and anaemia among children. History of anti-malarial drugs and bed net use and dried blood spots (DBS) were obtained from 1,271 (Asembo N = 327, Gem N = 451, Karemo N = 492) participants during the survey. For the study described here, 253 *P. falciparum* smear-positive samples, 61 in Asembo, 97 in Gem and 95 in Karemo, collected from participants <15 years old were available for genotyping of anti-malarial drug resistance molecular markers. In order to provide a temporal description in drug resistance markers, the data from the 2007 survey in Asembo were compared with molecular data collected from samples obtained in a 2001 community cross-sectional survey conducted in Asembo area in children < five years old as part of a randomized controlled trial of ITNs between 1996 and 2001 [17,34,35].

ITN use has been high in the Asembo and Gem areas since 1997 and 1999, respectively, when nets were first distributed to every household as part of an ITN efficacy trial. ITNs were introduced in Karemo in 2004 and scaled up in 2006 as part of a mass campaign to distribute free bed nets to children under the age of five. At the time of the 2007 survey, the parasitaemia prevalence in participants <15 years old was different among the three study areas: 35.8% (95% confidence interval (CI) 29.0-43.0%) in Asembo, 45.4% (95% CI 39.3-51.6%) in Gem and 50.3% (95% CI 43.3-57.2%) in Karemo (KEMRI/CDC, unpublished data). In the 2001 survey, the prevalence of parasitaemia in children < five years old was 34% in the Asembo area [34]. The entomologic inoculation rate (EIR) was estimated at four infective bites per person per year in Asembo and Gem and 20 infective bites per person per year in Karemo in the 2007 survey (KEMRI/CDC, unpublished data) and at 1.3 infective bites per person per year in Asembo area in 2001 [36].

In 1998, SP replaced CQ as first-line treatment policy for uncomplicated malaria in Kenya. National policy change from SP to AL was officially announced in 2004, although AL was not used in the study areas until mid-2006. AQ, the second-line drug during 1998–2004, temporarily became the first-line drug policy for treatment of uncomplicated malaria prior to the implementation of AL in 2006 [37,38]. SP remains the recommended drug for intermittent preventive treatment for malaria in pregnancy (IPTp) in the country. In addition, cotrimoxazole (CTX, trimethoprim-sulphamethoxazole) was commonly used for first line treatment of acute respiratory illnesses and also for prevention of opportunistic infection as a component of HIV care in western Kenya. CTX, a common antifolate antimicrobial, has similar anti-malarial properties to SP, by inhibiting *dhps* enzyme in the folic acid biosynthetic pathway.

This study was approved by the Ethical Review Committee of KEMRI, Nairobi, Kenya, the Institutional Review Board of Michigan State University, and the Institutional Review Board of CDC, Atlanta, Georgia. Written informed consent was obtained from parents or legal guardians of children and written assent was also obtained from the participants between 13 and 18 years of age.

### Laboratory methods

#### DNA extraction

Genomic DNA was purified from DBS using the commercial DNA purification QIAamp® blood mini-kit (Qiagen Inc, Valencia, CA, USA).

#### Genotyping of mutations in drug resistance genes

SNPs at *dhfr-*50, 51, 59, 108, 164, and *dhps-*436, 437, 540, 581, 613 were detected using published pyrosequencing procedures [39]. *Pfcrt* codons 72, 73, 74, 75, 76*, pfmdr1* codons 86, 184, 1034, 1042, 1246 and ambiguous results at *dhfr* and *dhps* genes were examined using direct sequencing [40].

### Pyrosequencing

Primary and secondary (nested) polymerase chain reaction (PCR) primers and pyrosequencing primers were synthesized at the CDC Biotechnology Core Facility, Atlanta, USA. Wild type and mutant positive controls and no DNA template negative controls were included in all experiments. Samples were run in pyrosequencing 96-well plates on PyroMark ID (Biotage AB, Uppsala, Sweden) and SNPs were called using the PyroMark ID 1.0 SNP software (Biotage AB, Uppsala, Sweden) [39].

### Direct sequencing

Nested PCR sequence procedures were used [40] and product sequences were read using Big Dye Terminator v3.1 cycle sequencing kit on iCycler thermal cycler (Bio-Rad, Hercules, CA, USA). Dye terminators were cleaned by precipitating reactions in 70% ethanol and rehydrating in 10 μL HiDi formamide. Reactions were sequenced on the 3130 ABI Genetic Analyzer (ABI Prism, Foster City, CA, USA). The predominant sequence in each sample was called based on the highest peak for each SNP.

#### *Pfmdr1* gene copy number

Copy number amplification was performed on the samples that were single infection as determined by the highly polymorphic, neutral microsatellite (MS) marker Poly-α [41]. Amplification of *pfmdr1* was detected using real-time PCR (Stratagene MX3005P; Agilent Technologies, Santa Clara, CA) using published reaction conditions, primer sequences and probes labelled with 3′ black hole quencher (BHQ) and 5′6-carboxyfluorescein (FAM), for *pfmdr*1 or 5′ hexachlorofluorescein (HEX), for β-tubulin [25]. Each experiment included a standard curve with five-fold serial dilutions of 3D7, 3D7 calibrator (one copy), the positive controls Dd2 (three to four copies) and W2Mef (two copies), no DNA template control, and field samples run in triplicate. Copy number was calculated using the comparative ΔΔC_t_ method and rounded to the nearest integer. Samples were repeated if the threshold cycle (C_t_) value was above 32 or if the two-tailed 95% CI (determined from the individual replicate ΔΔC_t_ calculations around the copy number estimation) exceeded 0.4 [25,42].

### Genetic definitions

All SNPs genotyped were classified as wild type or mutant and analysed individually first. Genotypes related to SP resistance markers (*dhfr*, *dhps* and combined *dhfr/dhps*) were defined based on the criteria outlined by Kublin and colleagues [18]. The *dhfr* genotype reflected mutations in *dhfr*-51, 59 and 108, classified as wild type, single, double and triple mutants. The *dhps* genotype described mutations in *dhps*-437 and 540, categorized as wild type, single and double mutants. The combined *dhfr*/*dhps* genotype (mutations in *dhfr*-51, 59, 108, and *dhps*-437, 540) was classified as wild type, single, double, triple, quadruple, and quintuple mutants.

Haplotypes were also constructed for d*hfr* codons 50, 51, 59, 108, and 164 and *dhps* codons 436, 437, 540, 581, and 613 in the samples that were single infection based on the neutral MS marker, poly-α. The *dhfr/dhps* haplotypes offer information on mutant variants in parasite populations that cannot be assessed from the genotypes described above. *Pfcrt* haplotypes based on codons 72, 73, 74, 75, and 76 and *pfmdr1* haplotypes, including codons 86, 184, 1034, 1042, and 1246, were reported based on direct sequencing data*.* In addition, CQ and SP combined haplotypes were assembled based on *pfcrt-*76*, pfmdr1-*86*, dhfr-*51, 59,108*,* and *dhps-*437, 540 [43,44] in the samples that were single infection by poly-α. The CQ and SP combined haplotypes provide information on the genetic linkage of mutations in parasites carrying drug resistance to multiple drugs and also an insight into potential synergistic effects by the different drug resistance genes on the recovery of sensitive parasites to individual drug [44,45].

### Data and statistical analysis

Differences in participant characteristics among the three study areas in the 2007 survey were analysed using Chi-square or Fisher’s exact test (when expected cell counts were less than five) for categorical variables and analysis of variance (ANOVA) for continuous, normally distributed variables. Parasite density was log transformed. Anaemia was defined as haemoglobin level < 11 g/dL. Statistical comparisons in the prevalence of all SNP mutations in *dhfr, dhps, pfcrt and pfmdr1* genes and SP resistance genotypes in samples among the three areas and between the 2001 and 2007 surveys were made using Chi-square tests. All statistical tests were considered as independent for each molecular marker. Statistical significance was defined by a two-sided p-value <0.05. Data and statistical analyses were performed using SAS software version 9.2 (SAS Institute Inc., Cary, NC, USA).

## Results

### Characteristics of smear-positive participants at the 2007 survey by study area

Smear-positive participants included in this study from Asembo, Gem and Karemo were similar in sex and age distribution, proportion anaemic, presence of gametocytes, fever within two weeks prior to survey, and anti-malarial drugs taken for fever. Geometric mean parasite density was low and also similar among the three areas. However, bed net and ITN use differed significantly between the study areas (p <0.0001). Reported bed net (treated or untreated) use in Karemo, Gem and Asembo, respectively, was 20, 44 and 51%, while ITN use was 7, 31 and 49% (Table 1). In addition, reported fever in last 24 hours differed significantly among the three areas (p <0.01), 15% for Asembo, 21% for Gem and 36% for Karemo (Table 1). Notably, self-reported AL use was low in all study areas, ranging from 0 to 3% (Table 1).

**Table 1:** **Characteristics of** ***Plasmodium falciparum*** **smear-positive participants at the 2007 survey in western Kenya, by study area**

**Characteristic**	**Asembo (n = 61)**	**Gem (n = 97)**	**Karemo (n = 95)**	**p-value** ^**a**^
Male sex	32/61 (53)	47/96 (49)	43/91 (46)	0.75
Age, years				
≤5	24/61 (39)	40/97 (41)	40/95 (42)	0.94
6-15	37/61 (61)	57/97(59)	55/95 (58)	
Anaemic, Hb < 11 g/dL	24 (39)	33 (34)	46 (48)	0.12
Parasite density, geometric mean, per uL (95% CI)^b^	548 (351–935)	513 (374–750)	704 (488–1,091)	0.54
Gametocytes present	9/61 (15)	19/97 (20)	12/95 (13)	0.40
Fever in last two weeks	31/61 (51)	62/95 (64)	47/95 (50)	0.088
Fever in last 24 hours	9/59 (15)	20/95 (21)	34/95 (36)	0.0083*
Drug taken for fever^c^				
Coartem	1/31 (3)	1/61 (2)	0/47 (0)	--
Sulphadoxine-pyrimethamine	4/31 (13)	2/61 (3)	2/47 (4)	0.20
Antifolate^d^	7/31(23)	4/61 (7)	3/47 (6)	0.055
Chloroquine	2/31 (7)	3/61 (5)	0/47 (0)	--
Amodiaquine	4/31 (13)	5/61 (8)	2/47 (4)	0.37
Other (non-anti-malarial)	24/31(77)	38/61 (62)	28/47 (60)	0.24
Bed net usage				
Any^e^ (treated or untreated)	31/61 (51)	42/95 (44)	19/95 (20)	<0.0001*
ITN^f^	30/61 (49)	29/95 (31)	7/95 (7)	<0.0001*

**Note.** Data are proportion (%) of *P. falciparum* smear-positive participants with molecular data, unless otherwise indicated.

Hb, haemoglobin; CI, confidence interval; ITN, insecticide-treated bed net.

^a^Derived from overall Chi-square or Fisher’s exact (where expected cell counts were below 5) test for categorical variables or analysis of variance (ANOVA) for continuous, normally distributed variables.

^b^Asexual parasite density and the presence of gametocytes were detected by microscopy. Parasite density was log transformed prior to statistical analysis.

^c^Self-reported drug use was asked for participants who reported being febrile in the two weeks prior to the survey; participant may have taken multiple drugs to treat fever.

^d^Sulphadoxine-pyrimethamine or Septrin (cotrimoxazole). ^e^Reported sleeping under a bed net the night prior to survey.

^f^ITN use among participants who reported sleeping under a bed net the night prior to survey.

*P <0.05, statistically significant.

### Prevalence of drug resistance molecular markers

#### At the 2007 survey

Since there were differences in the age distributions between the 2001 and 2007 surveys, the molecular profiles in children < five and participants between five and fifteen years of age were compared in 2007. No significant differences in molecular profiles were observed between the different age groups (Additional file 1) and therefore, subsequent comparisons between 2001 and 2007 were not adjusted for age.

**Figure 1:**
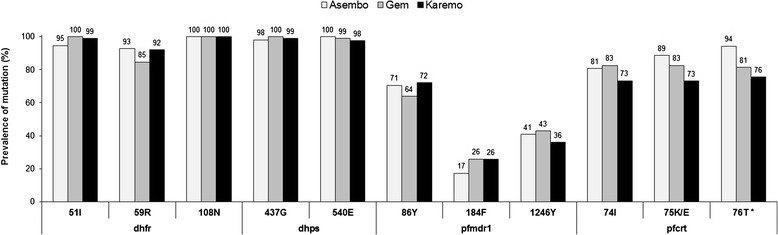
**Comparison of prevalence of point mutations among study areas.** Point mutations in *dhfr-*51, 59, 108, *dhps*-437, 540*, pfmdr1-*86, 184, 1246 and *pfcrt*-74, 75, 76 by study area. As few mutations were detected at *dhfr-*50, 164*, dhps-*436, 581, 613, *pfcrt-*72, 73 and *pfmdr1*-1034, 1042, the data are presented in the Results section, but not shown in this Figure. Statistical analysis performed using Chi-square tests. *p <0.05 difference in prevalence mutations between study areas.

CQ resistance mutations were highly prevalent; at least 73% of samples in all study areas harboured mutations at *pfcrt*-74**I**, 75**K/E**, or 76**T**, and mutations at *pfmdr1-*86**Y**, 184**F**, and 1246**Y** were detected at 69, 23 and 40%, overall, respectively (Figure 1). No samples carried mutations at *pfmdr1*-1034**C**, 1042**D** or *pfcrt*-72**S**. The prevalence of *pfcrt*-76**T** mutation, but not *pfmdr1*-86**Y**, differed significantly among study areas (p <0.02): 94% in Asembo, 81% in Gem and 76% in Karemo (Figure 1).

**Figure 2:**
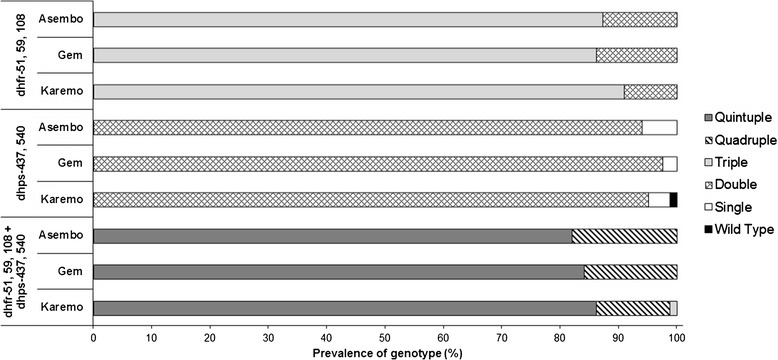
**Comparison of prevalence SP drug resistance genotypes among study areas.** *Dhfr*, *dhps*, and combined *dhfr/dhps* genotypes by study area. Statistical analysis performed using Chi-square tests. *p <0.05 difference in prevalence mutations between study areas.

The overall prevalence of key SP resistance mutations at *dhfr*-51**I**, 59**R**, 108**N** and *dhps*-437**G**, 540**E** exceeded 85% and there was no significant difference in these five SNPs by study area (Figure 1). All samples were wild type at *dhfr*-C50 and *dhps-*A581. Several mutations that are usually uncommon in Africa were observed in two samples at *dhfr-*164**L** (one sample in Gem, one in Karemo), three at *dhps-*436**A** (two samples in Gem, one in Karemo), and one at *dhps*-613**T** (Karemo). Combining all areas, the prevalence of the *dhfr* triple mutant (*dhfr* 51**I**/59**R**/108**N**) was 88%, *dhps* double mutant (*dhps* 437**G**/540**E**) was 96% and combined *dhfr/dhps* quintuple mutant (*dhfr* 51**I**/59**R**/108**N** + *dhps* 437**G**/540**E**) was 85%. These genotypes did not differ significantly by study area (Figure 2).

#### Comparison of 2001 and 2007 Asembo surveys

**Figure 3:**
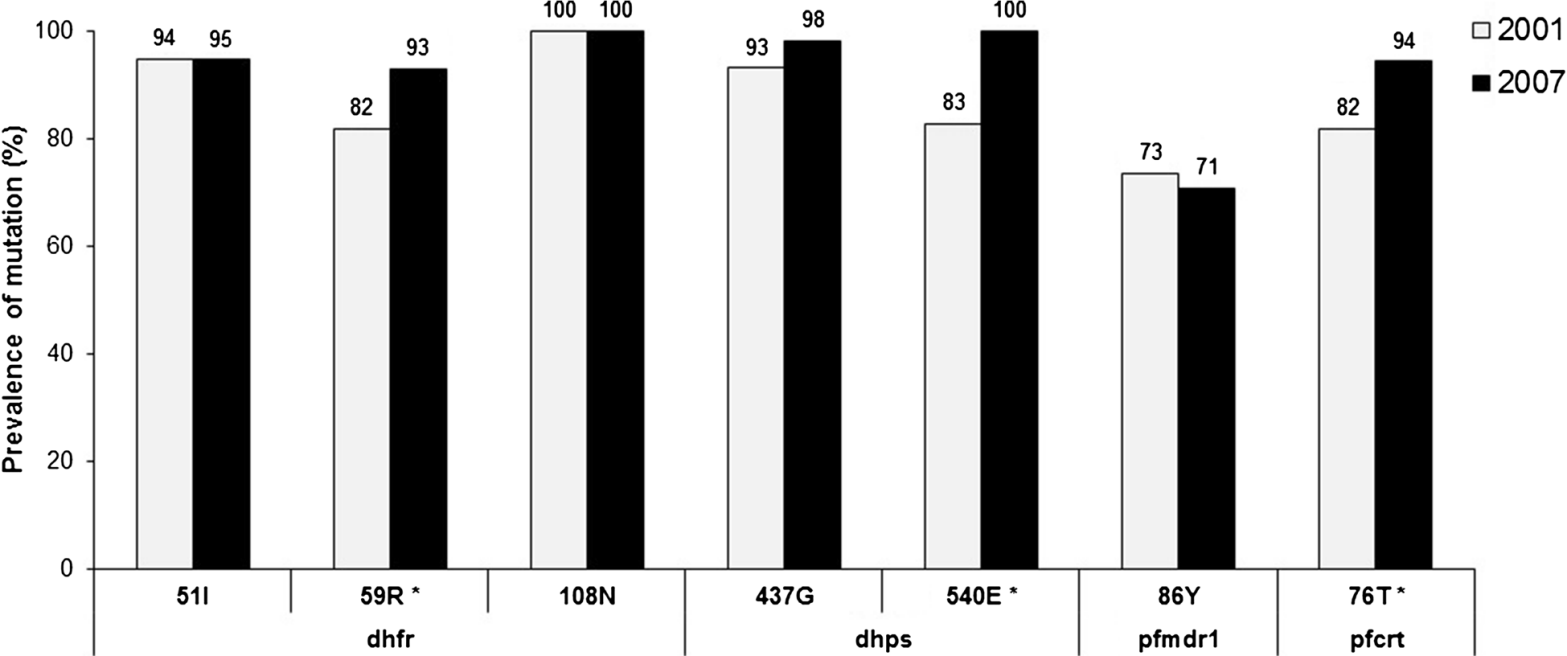
**Comparison of prevalence of point mutations in Asembo area between 2001 and 2007 surveys.** Point mutations in *dhfr* 51, 59, 108, *dhps* 437, 540*, pfmdr1-*86 and *pfcrt*-76 by study year. Statistical analysis performed using Chi-square tests. *p <0.05 difference in prevalence of mutations between 2001 and 2007.

**Figure 4:**
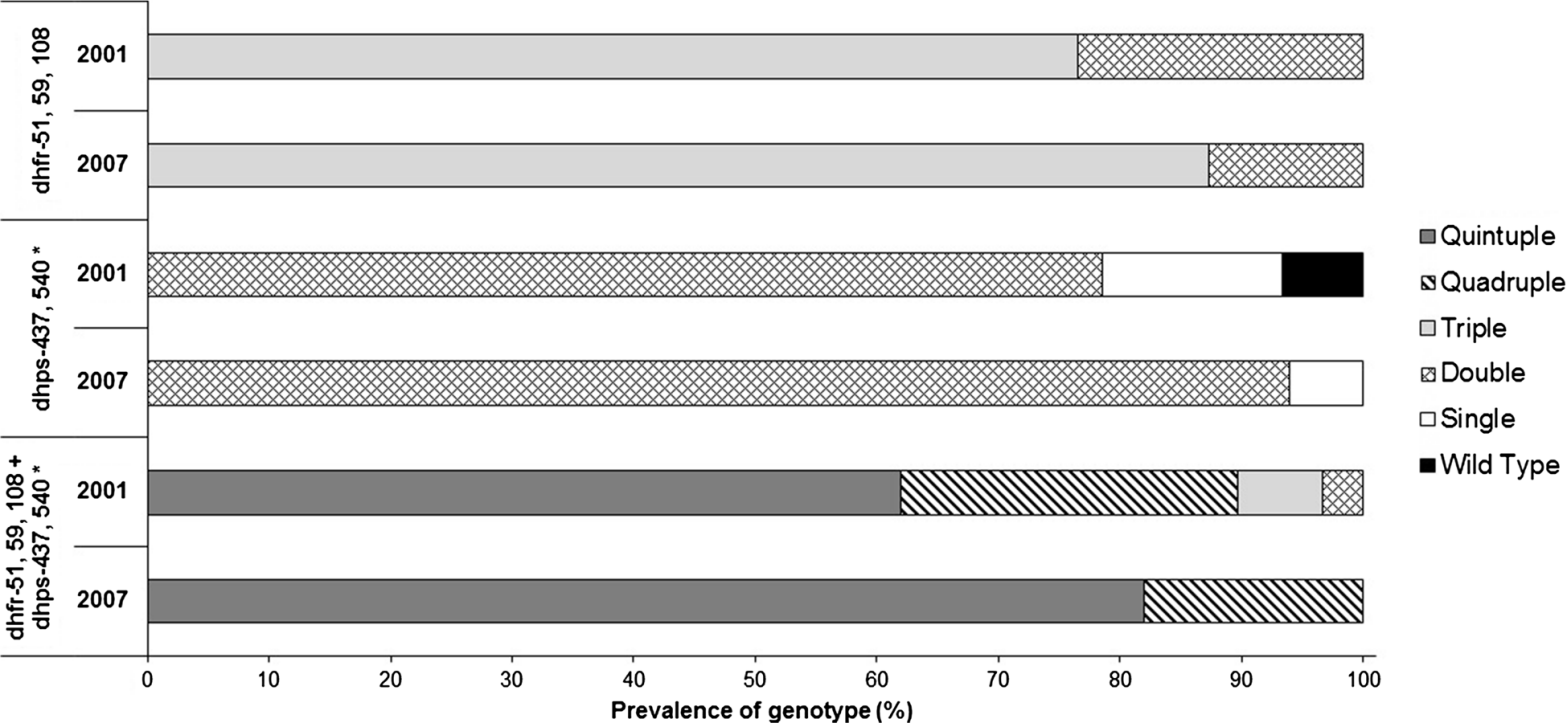
**Comparison of prevalence SP drug resistance genotypes in Asembo area between 2001 and 2007 surveys.** *Dhfr*, *dhps* and combined *dhfr/dhps* genotypes by study year. Statistical analysis performed using Chi-square tests. *p <0.05 difference in prevalence of mutations between 2001 and 2007.

Molecular marker data collected from a 2001 cross-sectional survey in Asembo area [17] were compared with the 2007 molecular data in Asembo only. The prevalence of mutations at *pfmdr1*-86**Y,***dhfr*-51**I**,108**N** and *dhps*-437**G** did not change between time points. A significant increase in mutations at *pfcrt*-76**T** from 82 to 94% (p <0.03), *dhfr*-59**R** from 82 to 93% (p <0.05), and *dhps*-540**E** from 83 to 100% (p <0.002) was observed from 2001 to 2007, respectively (Figure 3). Overall, the *dhps* and combined *dhfr/dhps* genotypes differed significantly between 2001 and 2007 (p <0.03). The *dhps* double mutant (*dhps*-437**G**/540**E**) increased considerably from 79% (2001) to 94% (2007) and the combined *dhfr/dhps* quintuple mutant (*dhfr* 51**I**/59**R**/108**N** + *dhps* 437**G**/540**E)** was 20 percentage points higher in 2007 compared to the 2001 prevalence of 62% (Figure 4). *Pfmdr1* gene copy number was determined in the samples that were single infection determined by the MS marker Poly-α at years 2001 and 2007. All samples were single copy for *pfmdr1*.

### Haplotypes of CQ, SP, and CQ and SP markers combined

#### Haplotypes of CQ resistance markers in the 2007 survey

The results of *pfcrt* and *pfmdr1* haplotypes are presented by study area (Tables 2 and 3). For *pfcrt,* 11 different haplotypes were observed: eight in Asembo, six in Gem and seven in Karemo. The majority of samples in all study areas were triple mutant type for *pfcrt-*74, 75, and 76, haplotype C_72_V_73_**I**_**74**_**E**_**75**_**T**_**76**_ (75% in Asembo, 80% in Gem, 69% in Karemo). The haplotype harbouring an alternative mutant codon **K** at 75, C_72_V_73_**I**_**74**_**K**_**75**_**T**_**76**_, was only detected in one sample from Asembo. Importantly, in Gem and Karemo, 15 and 21% of samples, respectively, were wild type for all five *pfcrt* codons (C_72_V_73_M_74_N_75_K_76_) while substantially fewer samples from Asembo were the wild type haplotype (4%). The remaining eight haplotype variants carrying either single mutation or double mutations at different codons were present from 1 to 8% in three study areas.

**Table 2:** ***Pfcrt*** **haplotypes at the 2007 survey in western Kenya, by study area**

***pfcrt*** **haplotype** ^**a**^	**72**	**73**	**74**	**75**	**76**	**No. mutations**	**Asembo, N (%)**	**Gem, N (%)**	**Karemo, N (%)**
							**(n = 52)**	**(n = 86)**	**(n = 89)**
Wild type	C tgt	V gta	M atg	N aat	K aaa				
**Mutant**	**S agt**		**I att**	**E gaa**	**T aca**				
	**S tct**			**K aaa**					
	C	V	M	N	K	0	2 (4)	13 (15)	19 (21)
	C	V	**I**	N	K	1	0 (0)	1 (1)	2 (2)
	C	V	M	**E**	K	1	0 (0)	1 (1)	0 (0)
	C	V	M	N	**T**	1	3 (6)	1 (1)	1 (1)
	C	V	M	**K**	**T**	2	4 (8)	0 (0)	0 (0)
	C	V	M	**E**	**T**	2	1 (2)	0 (0)	3 (3)
	C	V	**I**	N	**T**	2	1 (2)	0 (0)	2 (2)
	C	V	**I**	**K**	K	2	0 (0)	1 (1)	0 (0)
	C	V	**I**	**E**	K	2	1 (2)	0 (0)	1 (1)
	C	V	**I**	**K**	**T**	3	1 (2)	0 (0)	0 (0)
	C	V	**I**	**E**	**T**	3	39 (75)	69 (80)	61 (69)

**Note.** No. = number. Wild type amino acids are shown in normal font, while mutated amino acids are depicted in bold font. ^a^The construction of the *pfcrt* haplotype included codons 72, 73, 74, 75, and 76. All isolates coded for wild type C72 and V73.

**Table 3:** ***Pfmdr1*** **haplotypes at the 2007 survey in western Kenya, by study area**

***pfmdr1*** **haplotype** ^**a**^	**86**	**184**	**1034**	**1042**	**1246**	**No. mutations**	**Asembo, N (%)**	**Gem, N (%)**	**Karemo, N (%)**
							**(n = 47)**	**(n = 75)**	**(n = 70)**
Wild type	N aat	Y tat	S agt	N aat	D gat				
**Mutant**	**Y tat**	**F ttt**	**C tgt**	**D gat**	**Y tat**				
	N	Y	S	N	D	0	5 (11)	10 (13)	9 (13)
	N	Y	S	N	**Y**	1	2 (4)	8 (11)	2 (3)
	N	**F**	S	N	D	1	5 (11)	6 (8)	7 (10)
	**Y**	Y	S	N	D	1	16 (34)	20 (27)	22 (31)
	N	**F**	S	N	**Y**	2	1 (2)	3 (4)	1 (1)
	**Y**	**F**	S	N	D	2	2 (4)	6 (8)	7 (10)
	**Y**	Y	S	N	**Y**	2	16 (34)	17 (23)	19 (27)
	**Y**	**F**	S	N	**Y**	3	0 (0)	5 (7)	3 (4)

**Note.** No. = number. Wild type amino acids are shown in normal font, while mutated amino acids are depicted in bold font.

^a^ The construction of the *pfmdr1* haplotype included codons 86, 184, 1034, 1042, and 1246. All isolates coded for wild type S1034 and N1042.

A total of eight haplotypes were found for *pfmdr1*, with all eight of these haplotypes present in Gem and Karemo but seven present in Asembo. The wild type haplotype N_86_Y_184_S_1034_N_1042_D_1246_ was detected in 11% of samples from Asembo and 13% of samples in Gem and Karemo. The predominant haplotypes in all three areas contained a single mutation at codon 86, **Y**_**86**_Y_184_S_1034_N_1042_D_1246_ (34% in Asembo, 27% in Gem, 31% in Karemo), or double mutations at codons 86 and 1246, **Y**_**86**_Y_184_S_1034_N_1042_**Y**_**1246**_ (34% in Asembo, 23% in Gem, and 27% in Karemo). The triple mutant at *pfmdr1-*86, 184 and 1246*,* haplotype **Y**_**86**_**F**_**184**_S_1034_N_1042_**Y**_**1246**_, was found in 7% of samples from Gem, 4% of samples from Karemo and, of note, no samples from Asembo. The remaining four haplotype variants with single mutation (codon 184 or 1246) or double mutations (codons 184 and 1246 or 86 and 184) were present from 1 to 11% in three areas.

#### Haplotypes of SP resistance markers in the 2007 survey

In total, eight different haplotypes were detected at the 2007 survey across all study areas. Four haplotypes were observed in Asembo, five in Gem and three in Karemo. Overall, approximately 72% of isolates in all three study areas were C_50_**I**_**51**_**R**_**59**_**N**_**108**_I_164_S_436_**G**_**437**_**E**_**540**_A_581_A_613_, carrying quintuple mutations at the key *dhfr* and *dhps* codons. Another major haplotype observed was the quadruple mutant C_50_**I**_**51**_C_59_**N**_**108**_I_164_S_436_**G**_**437**_**E**_**540**_A_581_A_613_ (19%, overall). The remaining quadruple haplotypes with different combination at five codons **(**I_51_R_59_N_108_A_437_E_540_) were present in four samples in three areas. In Gem, one sample contained a mutation at *dhps*-**A**436 in quintuple mutant and another sample had the *dhfr-***L**164 mutation in quadruple haplotype. The presence of the *dhfr* triple mutant without any additional mutations, C_50_**I**_**51**_**R**_**59**_**N**_**108**_I_164_S_436_A_437_K_540_A_581_A_613,_ was only observed in one sample from Karemo (Table 4).

**Table 4:** **Haplotypes for** ***dhfr*** **and** ***dhps*** **resistance markers combined at the 2007 survey in western Kenya, by study area**

**SP haplotype** ^**a**^	***dhfr 51***	***dhfr 59***	***dhfr 108***	***dhfr 164***	***dhps 436***	***dhps 437***	***dhps 540***	**No. mutations**	**Asembo, N (%) (n = 17)**	**Gem, N (%) (n = 32)**	**Karemo, N (%) (n = 25)**
Wild type	N aat	C tgt	S agc	I ata	S tct	A gct	K aaa				
**Mutant**	**I att**	**R cgt**	**N aac**	**L tta**	**A gct**	**G ggt**	**E gaa**				
	**I**	**R**	**N**	I	S	A	K	3	0 (0)	0 (0)	1 (4)
	N	**R**	**N**	I	S	**G**	**E**	4	2 (12)	0 (0)	0 (0)
	**I**	C	**N**	I	S	**G**	**E**	4	3 (18)	6 (19)	5 (20)
	**I**	**R**	**N**	I	S	**G**	K	4	0 (0)	1 (3)	0 (0)
	**I**	**R**	**N**	I	S	A	**E**	4	1 (6)	0 (0)	0 (0)
	**I**	**R**	**N**	I	S	**G**	**E**	5	11 (65)	23 (72)	19 (76)
	**I**	C	**N**	**L**	S	**G**	**E**	5	0 (0)	1 (3)	0 (0)
	**I**	**R**	**N**	I	**A**	**G**	**E**	6	0 (0)	1 (3)	0 (0)

**Note**. SP = sulphadoxine-pyrimethamine; No. = number. Wild type amino acids are shown in normal font, while mutated amino acids are depicted in bold font. All samples were single infection for the microsatellite marker poly-α.

^a^The construction of the SP haplotype included *dhfr*-50, 51, 59, 108, 164 and *dhps*-436, 437, 540, 581, 613. All samples coded for wild type C at *dhfr*-50, A at *dhps*-581, and A at *dhps*-613.

#### Comparison of haplotypes of CQ and SP resistance markers combined between 2001 and 2007

CQ and SP combined haplotypes, based on *pfcrt*-76, *pfmdr1*-86, *dhfr*-51, 59, 108, and *dhps-*437, 540, were constructed to determine if there is linkage between CQ resistance genes and the accumulation of SP resistance genes. The combined haplotypes were compared among study areas in 2007 and between 2001 and 2007. Overall, fewer haplotypes were observed at 2007 (seven haplotypes) compared to 2001 (23 haplotypes). In the 2007 survey, two novel haplotypes were observed: **T**_**76**_**Y**_86_**I**_**51**_**R**_**59**_**N**_**108**_A_437_**E**_**540**_ (six mutations) in Asembo and K_76_N_86_**I**_**51**_**R**_**59**_**N**_**108**_**G**_**437**_K_540_ (four mutations) in Gem. Most notably, the prevalence of the septuple mutant haplotype (**T**_**76**_**Y**_**86**_**I**_**51**_**R**_**59**_**N**_**108**_**G**_**437**_**E**_**540**_) increased from 28% in 2001 to 39% in 2007 in the Asembo area. It was also noted that 48 and 41% of parasite isolates from Gem and Karemo, respectively, carried the septuple mutant haplotype in 2007 (Table 5).

**Table 5:** **Haplotypes for CQ and SP resistance markers combined at the 2001 (Asembo) and 2007 (Asembo, Gem, and Karemo) western Kenya surveys**

**CQ and SP haplotype** ^**a**^	***crt 76***	***mdr1 86***	***dhfr 51***	***dhfr 59***	***dhfr 108***	***dhps 437***	***dhps 540***	**No. mutations**	Asembo 2001* N (%) (n = 111)	**Asembo 2007, N (%) (n = 18)**	**Gem 2007, N (%) (n = 31)**	**Karemo 2007, N (%) (n = 22)**
Wild type	K aaa	N aat	N aat	C tgt	S agc	A gct	K aaa					
**Mutant**	**T aca**	**Y tat**	**I att**	**R cgt**	**N aac**	**G ggt**	**E gaa**					
	K	N	**I**	C	**N**	A	K	2	1 (1)	0 (0)	0 (0)	0 (0)
	**T**	N	**I**	C	**N**	A	K	3	1 (1)	0 (0)	0 (0)	0 (0)
	**T**	**Y**	**I**	C	**N**	A	K	4	3 (3)	0 (0)	0 (0)	0 (0)
	**T**	**Y**	N	**R**	**N**	A	K	4	1 (1)	0 (0)	0 (0)	0 (0)
	K	**Y**	**I**	**R**	**N**	A	K	4	1 (1)	0 (0)	0 (0)	0 (0)
	**T**	N	**I**	**R**	**N**	A	K	4	2 (2)	0 (0)	0 (0)	0 (0)
	**T**	N	**I**	C	**N**	**G**	K	4	1 (1)	0 (0)	0 (0)	0 (0)
	**T**	N	N	**R**	**N**	**G**	K	4	2 (2)	0 (0)	0 (0)	0 (0)
	K	N	**I**	**R**	**N**	**G**	K	4	0 (0)	0 (0)	1 (3)	0 (0)
	K	N	**I**	C	**N**	**G**	**E**	4	1 (1)	1 (6)	1 (3)	0 (0)
	**T**	**Y**	**I**	**R**	**N**	**G**	K	5	2 (2)	0 (0)	0 (0)	0 (0)
	**T**	**Y**	**I**	C	**N**	**G**	K	5	2 (2)	0 (0)	0 (0)	0 (0)
	K	**Y**	**I**	**R**	**N**	**G**	K	5	1 (1)	0 (0)	0 (0)	0 (0)
	**T**	N	**I**	**R**	**N**	**G**	K	5	1 (1)	0 (0)	0 (0)	0 (0)
	K	**Y**	**I**	C	**N**	**G**	**E**	5	3 (3)	0 (0)	2 (7)	1 (5)
	**T**	N	**I**	C	**N**	**G**	**E**	5	7 (6)	0 (0)	3 (10)	1 (5)
	**T**	N	N	**R**	**N**	**G**	**E**	5	4 (4)	1 (6)	0 (0)	0 (0)
	K	N	**I**	**R**	**N**	**G**	**E**	5	3 (3)	0 (0)	0 (0)	1 (5)
	**T**	**Y**	**I**	**R**	**N**	**G**	K	6	4 (4)	0 (0)	0 (0)	0 (0)
	**T**	**Y**	**I**	**R**	**N**	A	**E**	6	0 (0)	1 (6)	0 (0)	0 (0)
	**T**	**Y**	N	**R**	**N**	**G**	**E**	6	5 (5)	1 (6)	0 (0)	0 (0)
	**T**	**Y**	**I**	C	**N**	**G**	**E**	6	11 (10)	2 (11)	1 (3)	3 (14)
	K	**Y**	**I**	**R**	**N**	**G**	**E**	6	1 (1)	0 (0)	0 (0)	3 (14)
	**T**	N	**I**	**R**	**N**	**G**	**E**	6	23 (21)	5 (28)	8 (26)	4 (18)
	**T**	**Y**	**I**	**R**	**N**	**G**	**E**	7	31 (28)	7 (39)	15 (48)	9 (41)

**Note**. CQ = chloroquine; SP = sulphadoxine-pyrimethamine; No. = number. Wild type amino acids are shown in normal font, while mutated amino acids are depicted in bold font. All samples were single infection for the microsatellite marker poly-α.

^a^The construction of the CQ-SP haplotype included *pfcrt*-76, *pfmdr1* -86, *dhfr*-51, 59, 108, and *dhps*-437, 540.

*Data are derived from a previous study [17].

## Discussion

The objective of this study was to assess the effect of changes in drug treatment policy and ITN scale-up on resistance markers for CQ, SP and lumefantrine. At the 2007 survey, only the prevalence of *pfcrt*-76**T** mutation differed among study areas. The prevalence of CQ and SP resistance markers increased from 2001 to 2007 despite drug policy change. No molecular marker evidence for lumefantrine resistance was detected.

In order to address the study objective, qualitative rankings of transmission intensity were determined based on the proxy measures of ITN use, EIR, years since ITN scale-up and malaria prevalence. Collectively, these measures indicate that in 2007 a higher level of transmission in Karemo (ITN scale-up in 2006, ITN usage 7%, EIR 20 and parasite prevalence 50%) than in Asembo and Gem (ITN scale-up in 1997 and 1999, ITN usage 31-49%, EIR 4 and parasite prevalence 36-45%). In Asembo, there was no significant change in parasite prevalence and EIR between 2001 and 2007, suggesting that the intensity of malaria transmission was similar between the two time points. In 1998, SP was introduced as first-line treatment for uncomplicated malaria, followed by AL in 2006. While these changes occurred in an uncontrolled manner, it is useful to describe how these broad programmatically induced shifts in policies may have affected molecular profiles of anti-malarial drug resistance in this study.

A previous study conducted in Malawi observed a return of wild type *pfcrt*-K76 molecular marker and CQ efficacy 12 years after CQ was withdrawn [46]. In Kenya, CQ was replaced by SP as the national first-line treatment policy in 1998. A study conducted in coastal Kenya from 1993 to 2006 reported that following the policy change to SP, the prevalence of wild type *pfcrt*-K76 also increased, although at a slower rate than in Malawi [47]. However, this study showed a significant increase in the prevalence of *pfcrt*-76**T** mutation from 76% in 2001 to 94% in 2007. Data from a study conducted between 1996 and 2001 in the same area of western Kenya [17] and this study (Table 1) indicate that CQ was still used in this study area despite policy change. The continued use of CQ after policy change, although limited, could account for the observed significant increase in prevalence of *pfcrt-*76**T** mutation. Between 2004 and 2006, AQ was used temporarily as the first-line drug for treatment of uncomplicated malaria prior to the implementation of AL in 2006 and self-reported use of AQ was also observed in the current study (Table 1). Previous reports showed that the *pfcrt* haplotype, **S**_72_V_73_M_74_N_75_**T**_76,_ is associated with AQ treatment failure [22,23]. It is possible that the continued increase in the prevalence of *pfcrt*-76**T** mutation over time could be partially driven by the drug pressure derived from the use of AQ even though all samples were wild type at *pfcrt* -C72 (Table 2). In contrast, the prevalence of *pfmdr1*-86**Y** mutation remained unchanged over time. The significant increase in prevalence of parasites harbouring *pfcrt-*76**T** mutation but not *pfmdr1*-86**Y** mutation over time further support that CQ and/or AQ use selects for mutations in *pfcrt* [19,21,22,48,49].

Similarly, significant increases in the prevalence of the *dhps* double mutant and the *dhfr/dhps* quintuple mutant genotypes were observed (Figure 4) from 2001 to 2007, as well as the emergence of mutations at *dhfr-*164**L**, *dhps-*436**A** and *dhps*-613**T** in several 2007 samples. In the haplotype analysis of 2007 samples, 72% overall contained quintuple mutant haplotype parasites, C_50_**I**_**51**_**R**_**59**_**N**_**108**_I_164_S_436_**G**_**437**_**E**_**540**_A_581_A_613_ (Table 4). The increased selection of SP drug resistance parasites over time could be attributed to several factors. First, although at government health facilities, AL replaced SP as the first-line treatment for uncomplicated malaria in children in mid-2006, SP was still being used in the study area through 2007 (Table 1). This may have resulted in sustained drug pressure on these markers. Second, as SP remains the recommended drug for IPTp in the country, it is possible that pregnant women could serve as infectious reservoir for sustaining and circulating SP resistance parasites [50]. Third, the high use of CTX as an antibiotic treatment for respiratory infections and for the prevention of opportunistic infections in HIV-infected individuals might have also selected for SP drug resistance markers. Therefore, it is necessary to monitor SP molecular markers for an extended period in both the general population and pregnant women after a drug policy change leading to reduced SP drug pressure on the whole population.

The selection of wild type *pfmdr1-*N86, N1042, D1246, *pfcrt-*K76 and mutant type of *pfmdr1-*184**F**, and increased *pfmdr1* gene copy number have been associated with decreased lumefantrine sensitivity [27-30,32]. From 2001 to 2007, the prevalence of the *pfmdr1*-N86 wild type remained statistically unchanged from 26.6 to 29.4% (Table 1), while a significant increase in the *pfcrt-*76**T** mutation was found. Multiple copies of *pfmdr1* were not detected in samples at either time point. All 2007 samples were wild type *pfmdr1-*N1042 while 23.3% carried the mutation *pfmdr1-*184**F** and 40% had *pfmdr1*-1246**Y,** although 2001 samples were not genotyped for these three codons. The unchanged wild type *pfmdr1*-N86 from 2001 to 2007 along with low usage of AL (Table 1) detected in this study and too soon after the policy switch to AL is not sufficient to conclude that drug pressure by lumefantrine might select for N86 sensitive parasites. The studies conducted in Mozambique and Uganda reported an increase in parasite population with both *pfcrt-*K76 and *pfmdr1*-N86 wild types five years after policy change to AL [27,51]. In addition, *Henriques et, al.* observed the selection of *pfcrt*-K76 and *pfmdr1*-N86 wild types on day 3 in their ACT efficacy trial (including AL arm) conducted in Mbita, southwestern border of Kenya [28]. The difference in the *pfcrt-*76 results between this study and the report from Mozambique, Uganda and Mbita of Kenya could be mainly due to continued use of CQ and AQ as observed by the self-reported anti-malarial drug use in this study population (Table 1). Continuous monitoring of both *pfmdr1-*86 and *pfcrt-*76 in addition to *pfmdr1-*184, 1042, 1246 and *pfmdr1* gene copy number will be essential in detecting the emergence of lumefantrine resistance.

In this study, the drug resistance profiles were also assessed within the context of scale up of ITNs. Theoretical models suggest that changes in malaria transmission intensity, such as by the use of vector control interventions, can indirectly influence the spread of drug resistance through three main clinical/epidemiological ‘mediators’. As proposed by Hastings and Watkins, the three mediators could regulate several effector variables: 1) the number of parasite clones per host, which, in turn, determines the intrahost dynamics and rate of sexual recombination; 2) the perceived threat of infection that affects the level of community/population drug use; or, 3) immunity against malaria, which can change levels of therapeutic drug use and the biomass (number of parasites) per infection [2]. Additionally, the relationship between transmission intensity and spread of drug resistance depends on whether resistance to a drug is encoded by single gene or by multiple genes [2]. The genetic basis of SP is considered monogenetic as SP are encoded by *dhps* and *dhfr* genes, respectively. Mathematical models predict that the monogenic-based SP resistance spreads faster in high-transmission areas compared to medium- and low-transmission areas [2]. In contrast, CQ resistance, encoded by two genes, *pfcrt* and *pfmdr1* [19,49], is multigenic and follows a concave curve such that the prevalence of resistance markers is highest in low- and high-transmission areas and lowest in areas of intermediate transmission [2,52].

This study observed the prevalence of the *pfcrt*-76**T** mutation differed among areas of varying transmission intensity in 2007 with the highest prevalence in the lowest transmission area (Asembo) and lowest prevalence in highest transmission area (Karemo) (Figure 1). In addition, haplotype analysis of *pfcrt* showed that fewer samples from Asembo were the wild type (C_72_V_73_M_74_N_75_K_76_) haplotype compared to Gem and Karemo (Table 2). However, there were no significant differences in the prevalence of *pfmdr1*-86**Y** mutation and SP *dhfr/dhps* quintuple mutant genotype among the three areas (Figures 1 and 2). There are several reasons why the prevalence data for both CQ and SP markers from this study do not fit the theoretical models described above. First, the similarity in prevalence of *pfmdr1*, *dhfr* and *dhps* markers among the three areas could be explained by the essentially medium to high transmission and small relative differences in transmission intensity between areas. As noted, the difference between an EIR of 4 (Asembo and Gem) and an EIR of 20 (Karemo) would have minimal impact on parasite population genetic structure, resulting in reduced ability to determine the relationship between transmission intensity and the prevalence of molecular markers for the three genes. It is also possible that the gene flow due to migration of human and mosquito populations between the areas or from other parts of Kenya, unmeasured in this study, might contribute to the lack of difference in prevalence of the *pfmdr1*, *dhfr* and *dhps* gene markers among the three areas. Second, the observed higher prevalence of *pfcrt-*76**T** mutation and loss of more wild type (C_72_V_73_M_74_N_75_K_76_) haplotype in Asembo compared to the other two areas are weak although notable. The lack of information of CQ and AQ use at the population level for these three study areas and small sample sizes in the reported CQ and AQ use for fever in this study, limited the ability to explore drug pressure, influenced by transmission intensity, on the observed higher prevalence of *pfcrt-*76**T** mutation and loss of *pfcrt* wild haplotype in Asmebo. However, MS data from another study using the same set of samples showed that the overall proportion of multiple clone infections remained high in 2007 but relatively lower in Asembo (88%) compared to Karemo (97%). The relatively lower proportion of multiple clone infections in Asembo might be associated with the occurrence of intrahost dynamics, in which resistant parasite clones bearing *pfcrt* molecular markers benefit from removal of co-infecting drug-sensitive clones on treatment of human host [2], resulting in high prevalence of *pfcrt-*76**T** mutation and loss of *pfcrt* wild haplotype.

In this study, haplotypes based on key SNPs in *pfcrt, pfmdr1, dhfr,* and *dhps* [44,45] were also further compared between 2001 and 2007 surveys and by areas in 2007. In 2001, 23 haplotypes were found which reduced to seven in 2007, with fewer unique haplotypes observed in 2007. The results suggest selection of haplotype variants of multiple drug resistance genes possibly by genetic recombination during the sexual reproduction stage in mosquitoes [53] and the selection pressure could reduce the parasite population size resulting in a more likely loss of rare haplotypes. More importantly, an increase in the septuple mutant (**K**_**76**_**Y**_**86**_**I**_**51**_**R**_**59**_**N**_**108**_**G**_**437**_**E**_**540**_) was detected from 28% in 2001 to 39% in 2007 (Table 5) in Asembo. In addition, in 2007, other two neighbouring areas, Gem and Karemo, showed the prevalence of the septuple haplotype mutant similar to Asembo. These results suggest a strong linkage among mutations of *pfcrt, pfmdr1, dhfr,* and *dhps* in the parasite population and a possible effect by the strong linkage among the resistance genes on preventing the recovery of sensitive parasites to individual drug. The increase in the septuple mutant haplotype is alarming as sustained selection pressure could promote an increase in the prevalence of septuple mutant parasites to the point where both CQ and SP resistance mutations eventually reach fixation in the parasite population.

This study had a few limitations. The qualitative ranking of transmission intensity based on proxies, such as parasite prevalence, is subject to wide variation over time. The geographic proximity of the three study areas and evaluation of the effect of transmission intensity on drug resistance at a single point may have also limited the ability to detect differences in the molecular profiles of drug resistance among the areas. The small number of samples analysed in this study might have also reduced the statistical power to detect differences in the prevalence of mutations in all the molecular markers among the areas. In addition, the data presented here were from samples collected in 2001 and 2007 and may not reflect the most recent prevalence of drug resistance markers in the study areas. Additional surveys are currently underway to genotype samples collected after 2007 from the same study areas in order to provide more recent estimates and analyse trends in molecular marker prevalence after a longer period of time after AL implementation. Finally, molecular data on the prevalence of artemisinin resistance markers at baseline and in more recent parasite samples from this area will be critically important.

## Conclusion

Molecular markers associated with lumefantrine resistance were not detected in 2007. More recent samples will be needed to detect any selective effects by AL. The prevalence of CQ and SP resistance markers increased from 2001 to 2007 in the absence of changes in transmission intensity. Among these mutations, in 2007, only the prevalence of *pfcrt*-76**T** mutation differed among study areas of varying transmission intensity, with the highest prevalence in Asembo. Resistant parasites were most likely selected by sustained drug pressure from the continued use of CQ, SP, despite drug policy change to AL, and mechanistically similar drugs such as AM and CTX. There was no clear evidence that difference in transmission intensity, as a result of ITN scale-up, influenced prevalence of drug resistance molecular markers.

## Additional file

Additional file 1:
**Comparison of prevalence of point mutations, by age.**
