# Supplementary material for: Assessment of molecular markers for anti-malarial drug resistance after the introduction and scale-up of malaria control interventions in western Kenya
Source: Malar J. 2015 Feb 14;14:75. doi: 10.1186/s12936-015-0588-4 (PMC4331436; doi:10.1186/s12936-015-0588-4)
Supplement: Additional file 1: — Comparison of prevalence of point mutations, by age. [file 12936_2015_588_MOESM1_ESM.docx]

# Additional file 1:

# Table 1 Comparison of prevalence of point mutations, by age

| **SNP** | **< 5 years, n/N (%)** | **5-15 years, n/N (%)** | **p-value** |
| --- | --- | --- | --- |
| *dhfr-*51 | 71/72 (99) | 158/161 (98) | 0.80 |
| *dhfr-*59 | 66/73 (90) | 144/162 (89) | 0.73 |
| *dhfr-*108 | 72/72 (100) | 163/163 (100) | N/A |
| *dhps-*437 | 71/71 (100) | 152/154 (99) | 0.47 |
| *dhps-*540 | 68/68 (100) | 144/147 (98) | 0.55 |
| *pfmdr1-*86 | 45/69 (65) | 101/143 (71) | 0.43 |
| *pfmdr1-*184 | 17/71 (24) | 34/147 (23) | 0.89 |
| *pfmdr1-*1246 | 28/64 (44) | 51/133 (38) | 0.47 |
| *pfcrt-*74 | 58/72 (81) | 119/154 (77) | 0.58 |
| *pfcrt-*75 | 59/72 (82) | 121/153 (79) | 0.62 |
| *pfcrt-*76 | 59/72 (82) | 126/154 (82) | 0.98 |
|  |  |  |  |

**Note.** SN, single nucleotide polymorphism.
